# Air Pollution, Pollen, and Indoor Exposures in Allergic Conjunctivitis: A Systematic Review

**DOI:** 10.3390/life16020271

**Published:** 2026-02-04

**Authors:** Clara Martinez-Perez, Ana Paula Oliveira

**Affiliations:** 1 Department of Physics of Condensed Matter, Optics Area, University of Seville, Reina Mercedes S/N, 41012 Seville, Spain; 2 Instituto Superior de Educação e Ciências de Lisboa (ISEC Lisboa), Alameda das Linhas de Torres, 179, 1750-142 Lisboa, Portugal; ana.oliveira@iseclisboa.pt; 3 Centro de Investigação, Desenvolvimento e Inovação em Turismo (CiTUR)—Polo Estoril, Avenida Condes de Barcelona, n.º 808, 2769-510 Estoril, Portugal

**Keywords:** allergic conjunctivitis, environmental exposures, air pollution, pollen, occupational risk, biomarkers, systematic review

## Abstract

Environmental exposures are increasingly recognized as important drivers of ocular surface inflammation, yet their combined contribution to the onset, exacerbation, and clinical burden of allergic conjunctivitis (AC) has not been comprehensively synthesized. This systematic review evaluated the evidence linking air pollutants, aeroallergens, and indoor or occupational exposures with allergic conjunctivitis. The review was conducted according to PRISMA 2020 and AMSTAR-2 guidelines and registered in PROSPERO (CRD420251162399). PubMed, Web of Science, and Scopus were searched from inception to 18 September 2025. Two independent reviewers screened studies, extracted data, and assessed methodological quality using the MINORS tool. Owing to substantial heterogeneity, findings were synthesized narratively. Twenty-nine studies were included, encompassing more than three million outpatient visits. Consistent associations were observed between particulate matter, nitrogen oxides, sulfur dioxide, carbon monoxide, and ozone with increased AC incidence and symptom severity, with variations by age, sex, and season. Pollen and air pollutants frequently acted synergistically. Indoor exposures were associated with increased risk in children, while occupational settings demonstrated exposure–response relationships. Experimental studies identified mechanisms involving epithelial barrier disruption, NF-κB activation, and thymic stromal lymphopoietin signaling. Overall, environmental exposures substantially contribute to allergic conjunctivitis and may inform improved prevention and personalized clinical management.

## 1. Introduction

Allergic conjunctivitis (AC) is a frequent ocular surface disease and one of the most common causes of ophthalmologic consultations worldwide. Its clinical manifestations include itching, redness, tearing, burning, photophobia, and mucous discharge, symptoms that considerably impair patients’ quality of life and productivity [1]. AC belongs to the broader group of allergic conjunctival diseases (ACDs), which also comprise perennial allergic conjunctivitis (PAC), seasonal allergic conjunctivitis (SAC), vernal keratoconjunctivitis (VKC), atopic keratoconjunctivitis (AKC), and giant papillary conjunctivitis (GPC) [2,3]. The disease spectrum ranges from mild, recurrent conditions such as SAC and PAC to severe, sight-threatening forms such as VKC and AKC, which can lead to keratoconus, cataracts, or retinal detachment [4,5].

Epidemiological evidence shows that AC prevalence is increasing worldwide. In the United States, up to 40% of the population report ocular allergic symptoms [6], with SAC and PAC affecting an estimated 15–25% of individuals [7,8,9]. In Europe, prevalence has reached nearly 50%, partly driven by the spread of highly allergenic species such as ragweed after 2009 [10]. In China, rapid industrialization and urbanization contributed to over 295 million cases of AC in 2020 [11]. Despite this substantial burden, AC remains underdiagnosed and undertreated, as many individuals perceive symptoms as normal until they receive specific treatment [12]. Comorbidity with asthma, rhinitis, and other atopic conditions is common, with ocular allergy affecting 40–80% of these subgroups [13,14].

Environmental exposures are central drivers of AC. The ocular surface is directly exposed to the atmosphere and therefore susceptible to allergens such as pollen, dust, and animal dander [15]. Ambient air pollution, recognized as a major contributor to systemic diseases including cardiovascular, respiratory, and neurological disorders, also threatens ocular health. The World Health Organization estimates that one in ten deaths in children under five years is attributable to air pollution [16]. Although the systemic consequences are well studied, ocular outcomes remain less investigated. Historically, photochemical smog in Los Angeles and Tokyo during the 1950s–1970s was already linked to “allergic eye syndromes” [17]. Since then, multiple pollutants, including particulate matter (such as PM_10_, PM_2.5_), diesel exhaust particles, nitrogen oxides (NO_x_), sulfur dioxide (SO_2_), carbon monoxide (CO), and ozone (O_3_), have been shown to trigger or exacerbate AC [18,19].

Pathophysiologically, AC is primarily mediated by IgE-driven hypersensitivity reactions, with mast cells and eosinophils releasing histamine, cytokines, and chemokines that sustain ocular inflammation [20]. Environmental pollutants can exacerbate these mechanisms by disrupting the epithelial barrier, prolonging allergen retention, and amplifying immune activation [6,21]. Moreover, the delayed or lagged effects of air pollutants complicates causal interpretation, as unmodeled inflammatory responses may bias results if not adequately modeled [21].

Overall, current evidence suggests that AC is not only a prevalent hypersensitivity disorder but also a sentinel condition for environmental health. However, the available literature remains fragmented across heterogeneous study designs, exposure indicators, and outcome definitions, limiting comparability and integrated interpretation. In particular, previous studies have rarely examined multiple environmental exposures jointly, their potential interactions (e.g., pollutants and pollen), or systematically linked epidemiological findings with mechanistic and experimental evidence.

To address these gaps, this systematic review was prospectively registered and conducted in accordance with PRISMA 2020 and AMSTAR-2 guidelines. We aimed to provide a comprehensive and methodologically rigorous synthesis of epidemiological, clinical, and experimental evidence on environmental determinants of allergic conjunctivitis, integrating population-level associations with mechanistic insights. By consolidating this evidence, the review seeks to inform future research priorities, support environmental risk-based prevention strategies, and contribute to more personalized clinical management and public health decision-making.

## 2. Materials and Methods

### 2.1. Research Question and PICOS Framework

**Figure 1:**
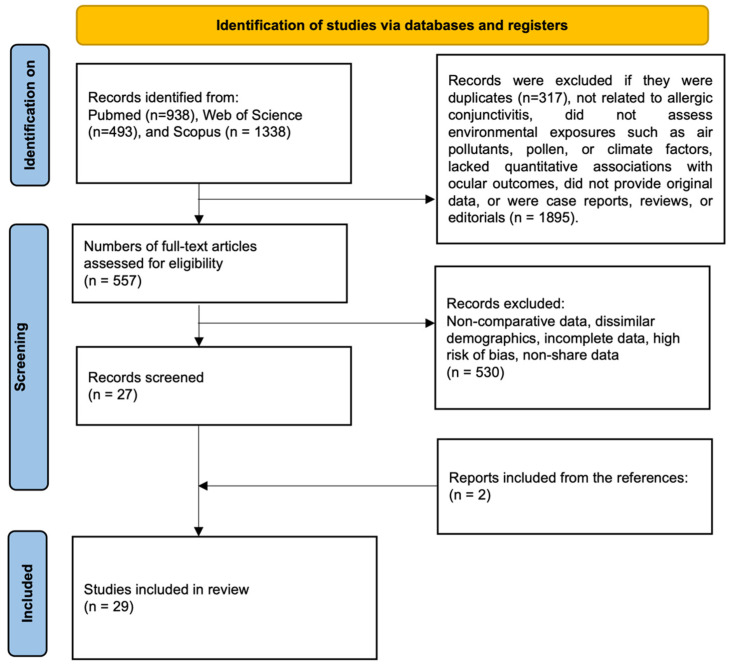
PRISMA flow diagram of study selection.

This systematic review was registered in PROSPERO (registration number: [CRD420251162399]) and conducted according to PRISMA 2020 guidelines [22] and AMSTAR-2 [23] methodological standards (Figure 1). A completed PRISMA checklist is provided as Supplementary Material (Supplementary File S1). The final literature search was completed on 18 September 2025. The research question was formulated using the PICOS framework to ensure methodological rigor and clinical relevance. Specifically, we examined whether environmental exposures, such as air pollutants, aeroallergens, meteorological variables, and occupational or indoor risk factors (Intervention/Exposure) are associated with the onset, exacerbation, or clinical burden of allergic conjunctivitis (Population), compared with unexposed groups or periods of lower exposure (Comparator). Eligible outcomes included incidence or prevalence of allergic conjunctivitis, frequency of outpatient visits, symptom severity, biomarker expression, and experimental evidence of immunological or pathophysiological mechanisms (Outcomes). All study designs providing quantitative or comparative data were considered (Study design), including ecological and case-crossover epidemiological studies, cross-sectional surveys, registry analyses, and experimental laboratory and animal models. Subgroup analyses considered exposure type (air pollution, pollen, indoor/occupational factors), geographic and demographic context, and study design as potential sources of heterogeneity. Through this comprehensive approach, the review aimed to synthesize evidence on environmental determinants of allergic conjunctivitis and to provide insights for clinical management, public health policies, and future research priorities.

### 2.2. Eligibility Criteria

Studies were excluded if they met any of the following criteria: (i) case reports or single-patient descriptions lacking generalizable outcomes; (ii) review articles (systematic or narrative), editorials, letters, or conference abstracts without full data; (iii) duplicate publications derived from the same dataset or experimental series; or (iv) studies judged to present insufficient methodological detail or high risk of bias. Additional exclusions included reports that did not assess environmental exposures (air pollutants, aeroallergens, meteorological variables, or occupational/indoor factors), did not provide comparative or quantitative data linking exposures to allergic conjunctivitis outcomes, or lacked original data or presenting incomplete information that prevented meaningful synthesis.

### 2.3. Information Sources

A comprehensive and systematic literature search was conducted using three major electronic databases: PubMed, Web of Science, and Scopus, without restrictions on publication date or language. To maximize coverage across the thematic domains of allergic conjunctivitis, environmental exposures (including air pollutants, aeroallergens, meteorological variables, and occupational or indoor factors), and associated health outcomes, the reference lists of all eligible articles were also manually screened to identify additional relevant studies not captured in the initial database search.

### 2.4. Search Methods for Identification of Studies

The search strategy combined controlled vocabulary and free-text terms related to allergic conjunctivitis and environmental exposures. Keywords encompassed descriptors of ocular allergy (seasonal, perennial, vernal, atopic) and environmental domains (air pollution, particulate matter (PM), pollen, aeroallergens, ultraviolet (UV) radiation, climate-related factors, and general environmental exposures). Full search strategies tailored to each database are provided in Supplementary File S2. Two reviewers independently assessed study eligibility at both the title/abstract screening and full-text review stages. Discrepancies were resolved through discussion and consensus. No language restrictions were applied, and studies published in languages other than English or Portuguese were translated and included when relevant data were available.

### 2.5. Data Extraction and Data Items

Two authors (A.P.O. and C.M.P.) independently extracted data from all eligible studies. For each included article, key characteristics were collected, including the first author’s name, year of publication, country or institutional setting, study design (epidemiological, clinical, experimental, or animal model), type of environmental exposure assessed (air pollutants, aeroallergens, meteorological variables, occupational or indoor factors), comparator group or condition, and primary outcomes. Discrepancies in data extraction or study inclusion were resolved through discussion and consensus, without the need for a third reviewer. Record management, including duplicate removal and tracking of study eligibility, was conducted using Microsoft Excel.

Primary variables extracted included environmental exposures (e.g., PM_2.5_, PM_10_, nitrogen dioxide (NO_2_), SO_2_, O_3_, carbon dioxide (CO_2_), pollen, aeroallergens, UV radiation, indoor/occupational factors), study population characteristics (e.g., age group, sex distribution, clinical diagnosis of SAC, PAC, VKC, or AKC), and methodological approach (e.g., time-series analysis, case-crossover design, cross-sectional survey, registry analysis, biomarker assessment, experimental models). These variables were selected a priori to allow identification and qualitative exploration of potential sources of heterogeneity related to exposure indicators, outcome definitions, and study design. Additional variables, such as sample size, outcome domains (incidence or prevalence of allergic conjunctivitis, outpatient visits, symptom severity, biomarker expression, mechanistic pathways), and reported limitations or conflicts of interest (e.g., industrial or pharmaceutical funding), were also recorded to support synthesis and highlight methodological heterogeneity across studies.

### 2.6. Data Synthesis and Assessment of Results

Given the heterogeneity of study designs, exposures, populations, and reported outcomes, a quantitative meta-analysis was not feasible. Therefore, a narrative synthesis approach was adopted. Heterogeneity arose primarily from three sources. First, exposure indicators and metrics varied widely across studies, including differences in pollutant or allergen type, averaging periods, spatial resolution, and lag structures, which limits comparability of effect estimates. Second, outcome definitions and ascertainment were inconsistent, ranging from allergic conjunctivitis–specific clinical diagnoses to broader conjunctivitis categories, healthcare utilization outcomes, symptom-based assessments, biomarker measurements, and mechanistic endpoints. These differences reflect fundamentally distinct outcome constructs that are not directly poolable. Third, heterogeneity was inherent to study design, encompassing ecological time-series and case-crossover analyses, cross-sectional surveys, clinical investigations, and experimental animal models, each characterized by distinct confounding structures, sources of bias, and inferential scope. These sources of heterogeneity affect both effect magnitude and direction, limiting interpretability and precluding meaningful statistical pooling.

Accordingly, results from individual studies were tabulated and synthesized by study design, type of environmental exposure, and main outcome domain (incidence or prevalence of allergic conjunctivitis, outpatient visits, symptom severity, biomarker expression, or mechanistic findings). Evidence was organized thematically into epidemiological, clinical, and experimental domains to facilitate qualitative comparison across populations, exposures, and methodological approaches. No statistical pooling, effect size estimation, or formal assessment of publication bias was performed.

### 2.7. Risk of Bias Assessment

The methodological quality and risk of bias of the observational studies included were independently evaluated by two reviewers using the Methodological Index for Non-Randomized Studies (MINORS), originally proposed by Slim et al. [24] (Supplementary File S4). The MINORS instrument assesses essential elements of study design, including the clarity of study aims, consecutive enrolment of participants, appropriateness of eligibility criteria, objectivity of outcome measurements, and adequacy of follow-up procedures.

For comparative studies, the total MINORS score ranges from 0 to 24, with studies categorized as very low quality (0–6), low quality (7–10), moderate quality (11–15), or high quality (16–24). Due to the heterogeneity of study designs, a formal risk-of-bias assessment was performed only for observational comparative studies (cross-sectional, cohort, or case–control designs) using the MINORS tool. Ecological time-series studies, case-crossover analyses, registry-based studies, and experimental or animal studies were included in the qualitative synthesis but were not subjected to formal risk-of-bias scoring. Any discrepancies between reviewers were resolved through discussion and mutual agreement.

## 3. Results

### 3.1. Study Selection

A total of 2769 records were initially retrieved from PubMed (n = 938), Web of Science (n = 493), and Scopus (n = 1338) (Figure 1). After removal of duplicates (n = 317), 2452 records were screened based on titles and abstracts, of which 1895 were excluded due to irrelevance to allergic conjunctivitis, lack of assessment of environmental exposures (e.g., air pollutants, pollen, or climate factors), absence of quantitative associations with ocular outcomes, non-original data, or being case reports, reviews, or editorials. Subsequently, 557 full-text articles were assessed for eligibility, and 530 were excluded due to non-comparative data, differing population characteristics, incomplete information, high risk of bias, or unavailability of shared data. In addition, two relevant studies were identified through manual reference screening. Ultimately, 29 studies met the inclusion criteria and were included in the qualitative synthesis [6,25,26,27,28,29,30,31,32,33,34,35,36,37,38,39,40,41,42,43,44,45,46,47,48,49,50,51,52].

### 3.2. Study Characteristics

Supplementary File S4 summarizes the key characteristics of the 29 studies included in this synthesis, which investigated the relationship between environmental exposures and allergic conjunctivitis across diverse contexts. Investigated exposures included air pollutants (PM_2.5_, PM_10_, NO_2_, SO_2_, O_3_, CO), pollen and other aeroallergens, occupational exposures (e.g., hazelnut and pear orchards), and indoor or lifestyle-related factors. Study designs ranged from large-scale epidemiological time-series analyses and population-based surveys to clinical investigations, laboratory-based biomarker assessments, and experimental animal models.

Research objectives were highly variable, encompassing short-term effects of ambient air pollution and pollen interactions on outpatient visits and symptom exacerbations, to mechanistic studies of PM–induced ocular allergy in murine models, and the identification of molecular biomarkers such as eotaxin-1, NF-κB activation, or immune-related long noncoding RNAs. Several studies also investigated geographical and demographic determinants of allergic conjunctivitis, highlighting vulnerable subgroups such as children, elderly individuals, males or females depending on pollutant type, and occupationally exposed populations.

Methodological approaches included ecological and case-crossover studies linking air quality and pollen data to health records, cross-sectional epidemiological surveys using questionnaires and clinical diagnoses, retrospective registry analyses covering millions of outpatient visits, and in vivo or in vitro experiments evaluating immunological pathways, cytokine responses, and ocular surface integrity. Both clinical and preclinical studies assessed seasonal allergic conjunctivitis as well as more severe forms, such as VKC and AKC, employing conjunctival biopsies, provocation tests, and tear biomarker assays.

Regarding analytical strategies, ten of the 29 included studies applied multipollutant or mixed-pollutant models, typically adjusting for co-pollutants such as PM_2.5_, PM_10_, NO_2_, O_3_, SO_2_, or CO within the same regression framework. This represents approximately one third of the included evidence. In contrast, the remaining studies relied on single-pollutant models, descriptive exposure assessments, or experimental designs. This imbalance in analytical approaches contributed to the methodological heterogeneity observed across the evidence base and should be considered when comparing results across studies.

Environmental factors investigated spanned traffic-related pollutants, biomass and occupational exposures, seasonal and geographical variations in pollen load, and emerging toxicants such as chlorinated paraffins. Reported outcomes included increased incidence of allergic conjunctivitis incidence, symptom exacerbations during pollution peaks, synergistic effects between pollen and pollutants, mechanistic insights into macrophage polarization and Th2-driven inflammation, and evidence supporting the effectiveness of air quality interventions.

Collectively, these studies underscore the multifactorial nature of allergic conjunctivitis in relation to environmental exposures. Large-scale epidemiological evidence indicates that high-level or combined environmental exposures are associated with measurable increases in disease burden; for example, a multicity study reported an approximately 8% increase in allergic conjunctivitis outpatient visits per interquartile increase in ambient ozone concentrations [50]. Proposed implications emphasized the importance of mitigating environmental risks through pollution control, allergen-conscious urban planning, protective behavioral strategies (e.g., eyewear, limiting outdoor exposure), and identification of novel therapeutic targets, highlighting both population-level risks and mechanistic pathways relevant to prevention and management.

**Figure 2:**
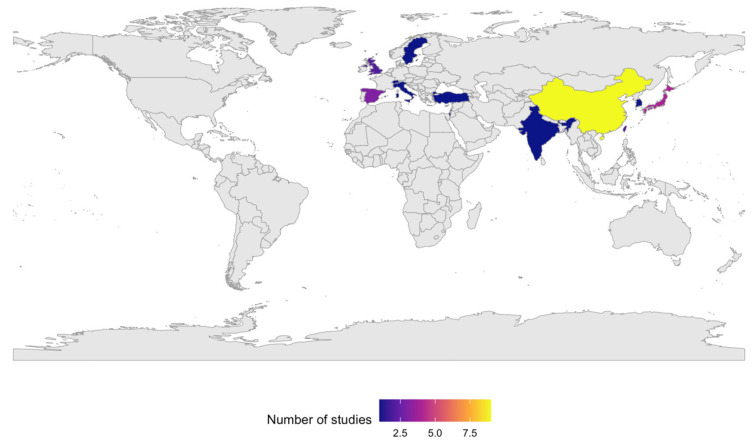
Geographic distribution of the included studies.

Overall, the 29 included studies spanned multiple cities and regions across several continents, including Asia, Europe, North America, South America, and the Caribbean. The evidence base comprised studies conducted in China, Japan, Taiwan, South Korea, India, Israel, Spain, the United Kingdom, Switzerland, Sweden, Italy, Turkey, the United States, Peru, and Trinidad and Tobago (Figure 2). Collectively, these studies encompassed more than three million cumulative individuals or outpatient visits across heterogeneous study designs, with publication dates ranging from 1997 to 2025.

The main characteristics and methodological quality of the included studies are summarized in Table 1.

### 3.3. Outcomes

#### 3.3.1. Environmental Factors and Exposures

Environmental exposures are central drivers of ocular allergy, especially AC. Epidemiological, clinical, and experimental evidence demonstrates that pollen, air pollution, indoor allergens, and occupational factors can act both independently and synergistically to increase disease burden. The 18 studies included in this review range from small occupational cohorts to national surveys and multicity time-series analyses, collectively demonstrating that AC as a sensitive indicator of environmental health challenges.

Occupational and aerobiological studies provide early and direct evidence of strong exposure–response relationships. Akcay Usta & Icoz [25] described ocular surface disease in hazelnut harvesters, linking pollen exposure to systemic inflammation and suggesting the value of blood biomarkers. Yanagisawa et al. [51] reported SAC in pear farmers exposed during artificial pollination, confirmed by eosinophil counts and IgE sensitization, with resolution after cromoglycate therapy. Calderón [28] identified Tipuana tipu pollen as a novel allergen, highlighting how local aerobiology can uncover new risks and inform urban greening policies. Chico-Fernández & Ayuga-Téllez [30,31] further demonstrated that common tree pollens and air pollutants interact synergistically to trigger conjunctivitis and rhinitis, emphasizing the need to integrate allergenicity into urban planning.

Large population-based time-series and registry studies provide quantitative evidence of age-related vulnerability to environmental exposures, although effect estimates are reported heterogeneously and cannot be directly pooled. In a nationwide registry study including over 3.2 million allergic conjunctivitis outpatient visits, Hong et al. [6] reported that individuals younger than 40 years were susceptible to multiple environmental factors (including NO_2_, O_3_, temperature, and humidity), whereas those aged 40 years and older showed more consistent associations primarily with NO_2_ exposure. Similarly, in a large time-series study from Urumqi (59,731 visits), Gui et al. [34] reported age-stratified relative risks, with significant associations for specific pollutants observed predominantly in young children and older adults, while intermediate adult age groups showed weaker or pollutant-specific effects. Although these age-specific estimates could not be pooled due to methodological and exposure heterogeneity, they consistently indicate greater relative vulnerability at the extremes of age. Liu et al. [39] showed lagged effects of particulate material and NO_2_, reinforcing temporal causality. Multicity studies from China Lu et al. [40] reported that even modest increases in PM_2.5_, PM_10_, NO_2_, SO_2_, and O_3_ were associated with elevated conjunctivitis risk, while Qiu et al. [50] focused on ozone, showing an 8% rise in AC visits per interquartile increase. These multicity studies confirm that associations between air pollutants and allergic conjunctivitis are robust and generalizable.

Japanese research refines the seasonal and mechanistic context. Mimura et al. [42] found PM_2.5_ associated with AC visits outside the pollen season, suggesting pollutant effects are more evident when allergen load is low. Mimura et al. [43] reported SPM and wind speed as the strongest predictors of AC across the year. Miyazaki et al. [44] provided prevalence estimates for SAC (45.4%), PAC (14.0%), AKC (5.3%), and VKC (1.2%), and linked traffic-related pollutants (NO_2_, NOx, PM_10_) with more severe keratoconjunctivitis forms, highlighting the environmental contribution to disease severity.

Regional and international studies show how local contexts shape ocular allergy burden. Ezinne et al. [33] found a prevalence above 40% in young adults, with associations to pollen, mites, cigarette smoke, and comorbid asthma and rhinitis, compounded by Saharan dust. Phiri et al. [46] (2025, Taiwan) identified indoor risk factors (furniture, carpets, mold, Der f 1) and outdoor PM_10_ as drivers of AC in preschool children, particularly boys. Gupta et al. [35] reported VKC prevalence above 1% in over 8000 children, with sunlight, dust, incense smoke, and seasonality as major triggers, and geographic variation with highest burden in the northern plains. These findings show how climate, culture, and housing shape exposure risks alongside pollutants.

Emerging contaminants expand the spectrum of environmental threats. Huang et al. [36] linked PM_2.5_-bound chlorinated paraffins (CPs) to allergic outcomes in over 130,000 children, with risks stronger in overweight individuals. Chlorinated paraffins are high-production-volume industrial chemicals increasingly detected in ambient air, household dust, and human biomonitoring samples, particularly in rapidly industrializing regions, raising concern about rising chronic low-level exposure in pediatric populations. These findings suggest new challenges for environmental ophthalmology beyond classical combustion-related pollutants.

**Figure 3:**
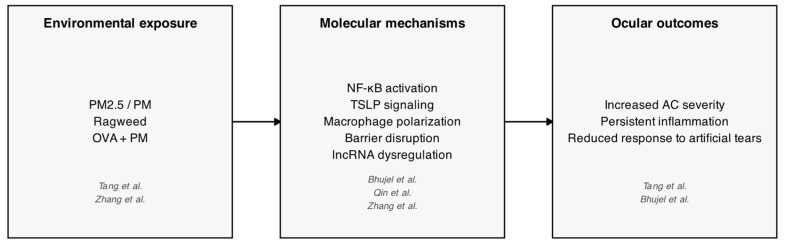
Mechanistic pathways linking environmental exposures to allergic conjunctivitis in experimental models [27,48,49,52].

Experimental models provide mechanistic evidence. Bhujel et al. showed that PM exposure worsens ovalbumin (OVA)-induced allergic eye disease in mice through NF-κB activation and barrier disruption. Tang et al. reproduced PM_2.5_-induced AC, with symptoms resistant to artificial tears. Qin et al. identified thymic stromal lymphopoietin (TSLP) signaling and macrophage polarization as key mediators of PM_2.5_-induced inflammation, while Zhang et al. profiled lncRNAs in ragweed-induced AC, suggesting novel immune regulators. Figure 3 provides a schematic synthesis of the exposure–mechanism–outcome pathways identified across experimental models.

Several consistent themes emerge across studies. Pollen and pollutants act synergistically, with O_3_, NO_2_, and PM amplifying allergic responses. Meteorological conditions, such as temperature and wind, modulate these effects, often intensifying risk during warm seasons. Vulnerable populations groups (including children, the elderly, and individuals with asthma or eczema) are disproportionately affected, with some sex-specific sensitivities (e.g., women to CO, men to NO_2_). Geographic context also influences risk: rural Indian children experience high burden of VKC, Caribbean populations face dust exposure and limited awareness, and East Asian cities are characterized by traffic-related air pollution–driven risk. Emerging environmental exposures, such as chlorinated paraffins, further expand concern to industrial chemicals.

Collectively, these findings frame AC as more than a localized hypersensitivity. Its high prevalence, clear environmental triggers, and measurable burden on the healthcare system establish AC as a sentinel condition for environmental health. Public health strategies should include stricter air quality regulations, allergen-conscious urban planting, improved housing standards, and targeted protection for vulnerable populations. Clinicians are encouraged to integrate environmental histories into routine diagnosis and management, while researchers should prioritize multipollutant exposure models and explore molecular mediators as potential therapeutic targets.

#### 3.3.2. Clinical Manifestations and Disease Burden

Population-based surveys and case-crossover analyses provide complementary insights into the epidemiology of ocular allergy and its environmental drivers. Singh et al. [47] analyzed data from NHANES III (1988–1994), showing that up to 40% of the U.S. population reported ocular allergy symptoms at least once over six years, the highest prevalence reported at that time. Ocular symptoms were more frequent than nasal ones in relation to pollen, animals, and dust, and older adults (>50 years) reported isolated ocular symptoms more often, whereas younger individuals experienced more combined ocular and nasal complaints. Skin prick testing confirmed strong associations with weed and pollen allergens, reinforcing the central role of aeroallergens in ocular allergy. This large dataset also underscored demographic and regional differences, with higher prevalence in the South and seasonal peaks in June–July.

Leonardi et al. [37] conducted one of the largest national surveys of ocular allergy in Europe, enrolling 3545 patients across 304 ophthalmology centers. They reported that SAC, PAC, VKC, and AKC together impose a substantial burden, yet allergy testing was performed in only 35% of cases, highlighting a diagnostic gap. The study revealed a mismatch between clinical practice and guidelines, with underuse of targeted therapies and overreliance on nonspecific medications. Importantly, it provided a detailed picture of triggers (pollen, dust, smoke, pollution) and comorbidities, offering a benchmark for clinical management across Europe.

Levanon et al. [38] advanced this evidence by linking VKC exacerbations to meteorological and air pollution exposures in a large population-based cohort of 6024 patients in southern Israel. Using a case-crossover design, they found that NO_2_, O_3_, and PM_10_ were strongly associated with exacerbations (ORs 1.9–2.3), alongside contributions from PM_2.5_, temperature, and solar radiation. Stratified analyses showed greater vulnerability in Bedouin children and socioeconomic subgroups, reflecting both biological and social determinants. These findings confirm that VKC flares are not solely seasonal phenomena but environmentally mediated events, sensitive to both pollutants and climatic extremes in semi-arid regions.

Together, these three studies illustrate the multilevel nature of ocular allergy research: national prevalence estimates defining scope (USA), large-scale clinical surveys exposing practice gaps (Italy), and environmental epidemiology pinpointing modifiable triggers (Israel). They converge on the conclusion that ocular allergy, including severe forms such as VKC, is highly prevalent, frequently underdiagnosed, and strongly influenced by both environmental exposures and healthcare practices.

#### 3.3.3. Pathophysiological Mechanisms and Biomarkers

Experimental and clinical studies have provided key evidence on the immunological mechanisms and environmental factors underlying AC. In one of the earliest histological investigations, Anderson et al. [26] performed conjunctival biopsies in patients with seasonal allergic conjunctivitis (SAC), demonstrating that the disease is fundamentally mast cell–dependent and, unlike VKC and AKC, lacks extensive granulocyte infiltration. MacLeod et al. [41] expanded on this, showing that mast cells are not only effector cells but also as cytokine reservoirs, with IL-4 release playing a central role in amplifying ocular inflammation. Subsequently, Eperon et al. [32] reinforced these clinical findings by identifying eotaxin-1 as a biomarker of SAC, highlighting its role in eosinophil recruitment via the C-C chemokine type 3 (CCR3) receptor. Complementing these studies, Nivenius et al. [45] conducted conjunctival provocation tests (CPT) in patients with AKC and SAC, observing both early and late responses, which confirmed the chronic involvement of IgE alongside T cell– and cytokine-mediated pathways.

Animal models have been particularly useful in exploring the interaction between environmental pollutants and allergic responses. Bhujel et al. [27], using a murine model of allergic eye disease (AED), demonstrated that co-exposure to PM and OVA enhances allergic inflammation through NF-κB activation, disrupting epithelial integrity and exacerbating systemic responses. Qin et al. [49] expanded these findings by establishing a reproducible PM_2.5_-induced AC model in BALB/c mice, identifying macrophage polarization toward the M1 phenotype and emphasizing TSLP as a central mediator of the inflammatory cascade. Similarly, Tang et al. [48] developed an acute murine AC model by direct PM_2.5_ instillation over 18 days, reproducing clinical signs such as eyelid edema, hyperemia, and tearing, along with conjunctival eosinophilic infiltration and goblet cell hyperplasia. This provided solid experimental proof that fine particles alone can induce a clinical phenotype analogous to human AC. Complementarily, Zhang et al. [52] applied a transcriptomic approach to a murine model of AC induced by ragweed pollen, identifying significant differences in the expression of mRNAs and lncRNAs associated with mucosal immunity. Their findings revealed the involvement of molecules such as Bpifa1 and Reg3g, as well as activation of inflammatory pathways including mitogen-activated protein kinase (MAPK) and signal transducer and activator of transcription (STAT), pointing to novel potential therapeutic targets for ocular allergy management.

Taken together, these eight studies span from the histological and molecular characterization of AC in humans to animal models that clarify the effects of environmental particles and specific allergens. The evidence converges on the central role of mast cells and eosinophils in SAC, the relevance of biomarkers such as eotaxin-1 and TSLP, and the capacity of PM_2.5_ to act both as an adjuvant and as a direct trigger of conjunctival inflammation. Furthermore, the integration of transcriptomics and conjunctival provocation models provides a more refined understanding of mucosal immunity, underscoring the need for therapeutic approaches aimed not only at symptomatic control but also at modulation of specific immune pathways.

## 4. Discussion

This systematic review highlights that environmental exposures, including air pollutants, aeroallergens, and indoor or occupational factors, are key drivers of AC. Across 29 studies, consistent associations were observed between PM, NO_x_, O_3_, and pollen peaks with increased incidence and severity of ocular allergy. Mechanistic investigations further confirmed that these exposures amplify IgE-mediated inflammation by disrupting the epithelial barrier, prolonging allergen retention, and triggering cytokine cascades. Together, these findings reinforce the notion that AC is not only a prevalent hypersensitivity disorder but also a sentinel condition reflecting the broader health impacts of environmental change.

**Figure 4:**
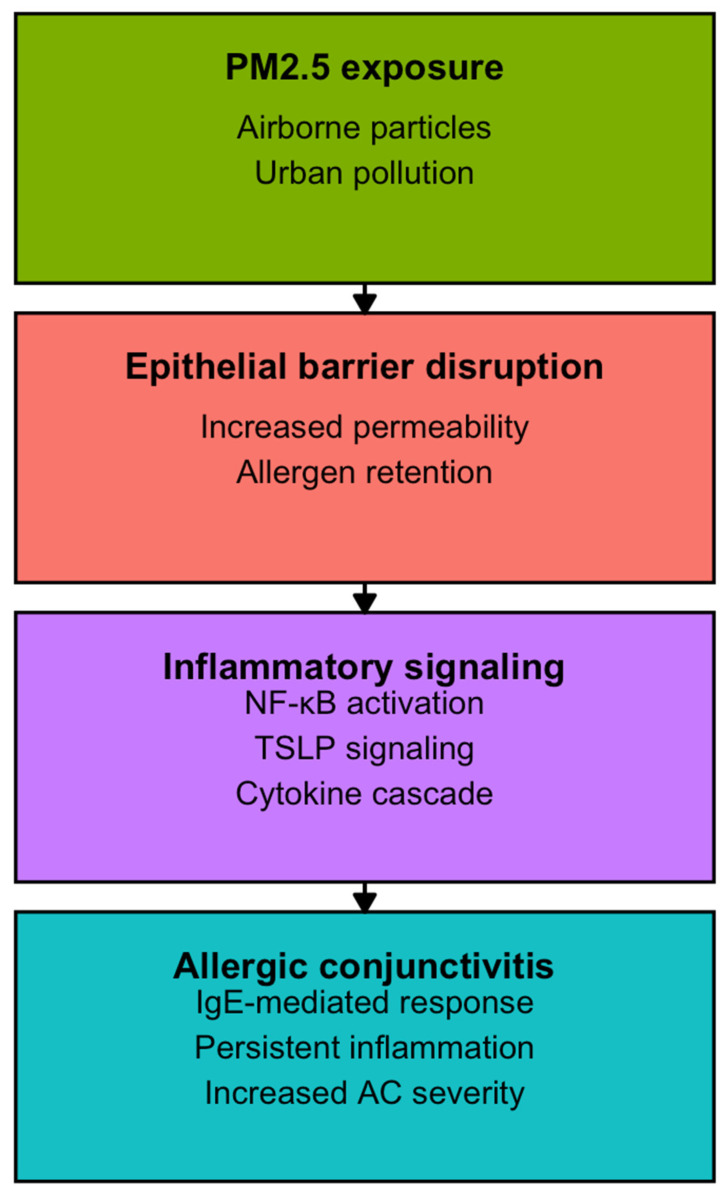
Conceptual pathways linking PM_2.5_ exposure to allergic conjunctivitis.

Our synthesis aligns with the report by Epstein et al. [53], which highlighted how climate change is reshaping aerobiology through longer pollen seasons, increasing allergenicity, and altered species distribution. While Epstein’s work was primarily predictive and focused on North America and Europe, our review consolidates empirical evidence from Asia, Africa, and Latin America, showing that these risks are already manifesting globally. Real-world support comes from registry analyses such as Hong et al. [6], which examined more than 3.2 million visits and identified NO_2_, O_3_, and temperature as consistent predictors, and by Gui et al. [34], which revealed pollutant-specific vulnerabilities by age and sex, corroborating the mechanisms anticipated by Epstein and colleagues. To integrate these epidemiological and experimental findings, Figure 4 provides a conceptual overview of the proposed pathways through which PM_2.5_ exposure disrupts epithelial barrier integrity, facilitates allergen penetration, and activates inflammatory signaling cascades, including NF-κB and TSLP. This framework links population-level associations with mechanistic evidence, illustrating how environmental exposures translate into amplified IgE-mediated ocular inflammation.

Mechanistic evidence strengthens these associations. Sedghy et al. [54] reviewed how diesel exhaust and ozone can increase pollen allergenicity by enhancing protein release and modifying epitopes, a phenomenon supported by studies from Chico-Fernández and Ayuga-Téllez [30,31], which demonstrated synergistic effects of tree pollens and air pollutants in urban environments. Across these studies, pollutants are consistently recognized as adjuvants that potentiate allergic responses; the main distinction lies in the perspective: molecular studies describe how allergenic potency is modified, whereas epidemiological work documents how such changes translate into population-level disease burden. Our findings also align with the mechanistic review by Upaphong et al. [55], which showed that PM induces oxidative stress, DNA damage, and mitochondrial dysfunction in ocular surface cells. This toxicological evidence is consistent with the delayed increases in AC after pollution peaks reported by Liu et al. [39] and the seasonal patterns observed by Mimura et al. [42,43], indicating that PM can trigger conjunctivitis even outside pollen season. Across these studies, pollutants are identified as direct ocular hazards; differences arise in exposure scale, with in vitro experiments examining controlled doses and composition, while population studies reflect heterogeneous exposures shaped by geography and meteorology.

From a clinical perspective, our synthesis echoes the observations of Ackerman et al. [56], who emphasized that AC is frequently underdiagnosed and undertreated despite its high prevalence. This underrecognition was also evident in a European survey by Leonardi et al. [37], where only 35% of patients underwent allergy testing despite substantial clinical burden. Both studies highlight the gap between prevalence and diagnosis, although Ackerman’s conclusions were based on practice surveys and guideline analysis, whereas Leonardi provided multicenter quantitative data.

The role of biomarkers further illustrates the bridge between mechanistic and clinical research. Shoji [57] demonstrated the diagnostic utility of tear and ocular surface biomarkers, such as eotaxin-2 and periostin, while our review identified upregulation of eotaxin-1, NF-κB, and TSLP in response to environmental exposures. Both strands emphasize the relevance of molecular signatures, yet their application differs: Shoji proposed standardized use in clinical diagnosis, whereas most studies in our synthesis treated biomarker assessment as exploratory. In this context, emerging biosensing technologies may play a key role in translating molecular biomarkers into clinical practice by enabling sensitive, rapid, and clinically adaptable detection of immunological and inflammatory signals. Recent advances in optically active two-dimensional nanomaterials, such as MoS_2_-based biosensing platforms, have demonstrated high sensitivity and versatility for biomolecular detection, supporting their potential applicability in future ophthalmic and immunological biomarker assessment and strengthening the translational link between mechanistic research and clinical evaluation [58]. This gap underscores the need to translate experimental findings into routine practice.

Animal models also provide compelling evidence of causality. Groneberg et al. [59] showed that OVA and ragweed models clarified mast cell–driven hypersensitivity, while more recent work by Bhujel et al. [27] demonstrated that PM exacerbates allergic eye disease through mast cell degranulation, cytokine release, and conjunctival apoptosis. These findings are consistent with those of Qin et al. [49] and Tang et al. [48], who reproduced PM_2.5_-induced AC in mice and highlighted the role of TSLP signaling, macrophage polarization, and goblet cell hyperplasia. Over two decades of research, earlier models emphasized allergens as primary triggers, whereas recent experiments establish pollutants as independent pathogenic agents, bridging mechanistic insights with clinical relevance.

Despite broad agreement, notable discrepancies remain. Large registry studies consistently implicate NO_2_, O_3_, and PM as major contributors to AC, yet their effects vary across demographic groups. For example, Gui et al. [34] identified stronger associations in women, infants, and the elderly, while Levanon et al. [38] reported heightened vulnerability among Bedouin children and socioeconomically disadvantaged populations in Israel. Biomarker responses are similarly heterogeneous, with some studies demonstrating marked upregulation of eotaxin or TSLP, while others report modest changes. Such differences likely reflect variations in pollutant composition, geographical context, and methodological approaches, reinforcing the need for standardized exposure and outcome measures. Nevertheless, several themes emerge consistently. Pollen and pollutants act synergistically to amplify ocular allergy, meteorological conditions such as temperature and wind often exacerbate risk, and vulnerable groups, including children, the elderly, and patients with asthma or eczema, are disproportionately affected. Emerging contaminants, such as chlorinated paraffins [36], expand concern to industrial chemicals, highlighting environmental threats beyond classical combustion products.

Several important limitations of the available evidence must be acknowledged. The marked heterogeneity observed across studies (related to differences in exposure assessment, outcome definitions and ascertainment, and study design) limited direct comparability and precluded quantitative synthesis. This heterogeneity encompassed the use of diverse environmental indicators (e.g., PM_2.5_, PM_10_, NO_2_, O_3_, pollen), exposure metrics and lag structures, as well as variability in outcome assessment, ranging from allergic conjunctivitis–specific diagnoses to broader conjunctivitis categories and from healthcare utilization outcomes to symptom-based, clinical, or biomarker measures. In addition, heterogeneity inherent to study design, including ecological time-series and case-crossover analyses, cross-sectional surveys, clinical investigations, and experimental animal models, introduced distinct confounding structures and potential sources of bias that should be considered when interpreting the findings. Many investigations relied on ecological or city-level exposure estimates, which increase the risk of exposure misclassification and restrict the ability to account for individual-level confounders such as socioeconomic status, pre-existing atopy, or access to care. Second, most studies were conducted in East Asia and Europe. Although a small number of studies originated from Latin American or Caribbean settings and other tropical or subtropical regions, these were limited in number and scope and did not provide systematic or regionally representative evidence. No eligible studies were identified from Africa. Overall, tropical and low- and middle-income regions remain underrepresented. This geographic imbalance likely reflects gaps in research coverage and data availability rather than selective inclusion and limits the global generalizability of the findings. Third, although real-world exposures occur as complex mixtures, only a minority of studies adopted multipollutant models, and few applied advanced mixture methodologies despite strong collinearity among pollutants. In addition, although multiple clinical subtypes of allergic conjunctivitis (SAC, PAC, VKC, and AKC) were included, structured comparisons across subtypes were not feasible, as most studies focused on a single subtype or did not report outcomes stratified by clinical phenotype. Finally, longitudinal biomarker studies and subtype-specific analyses (e.g., SAC/PAC vs. VKC/AKC) remain scarce, limiting our understanding of temporal relationships between exposures, molecular pathways, and clinical manifestations.

On the other hand, most experimental animal models included in this review employed short-term and relatively high-dose PM_2.5_ exposure protocols to induce allergic conjunctivitis. While these models are valuable for elucidating mechanistic pathways and establishing causality, they do not fully recapitulate the chronic, low-dose, and cumulative exposure patterns characteristic of real-world human environmental exposure. Consequently, their translational relevance for long-term disease development, adaptation processes, and exposure–response relationships may be limited. Future experimental studies should prioritize chronic and repeated low-dose exposure paradigms to better reflect environmentally relevant conditions and improve alignment with epidemiological evidence. A key strength of this review lies in its integrative scope, combining epidemiological, clinical, and experimental evidence to provide a comprehensive overview of environmental determinants of allergic conjunctivitis. By synthesizing findings across multiple exposure domains, including air pollutants, aeroallergens, meteorological variables, and indoor or occupational factors, this work moves beyond isolated exposure–outcome associations and offers a unified framework linking environmental change to ocular allergic disease. The prospective registration and adherence to PRISMA and AMSTAR-2 standards further enhance methodological transparency and reproducibility.

Looking forward, several priorities emerge for advancing the field. Future studies should promote standardized outcome definitions and harmonized exposure assessment protocols to facilitate comparability across multi-center and multi-country cohorts. Longitudinal designs integrating detailed clinical phenotyping with molecular and immunological biomarkers are particularly needed to validate mechanistic pathways such as epithelial barrier dysfunction and TSLP signaling in human biospecimens, thereby strengthening translational relevance. From an analytical perspective, the adoption of multipollutant and mixture-based modeling approaches will be essential to quantify interaction effects among pollutants, aeroallergens, and meteorological factors under real-world conditions. Expanding research on emerging contaminants, including industrial chemicals, is also warranted given evolving exposure profiles. Finally, interventional and implementation studies evaluating the real-world effectiveness of preventive strategies, such as air filtration, exposure reduction policies, and protective eyewear, will be critical for translating mechanistic and epidemiological evidence into clinical practice and public health action.

## 5. Conclusions

Allergic conjunctivitis represents the intersection between environmental exposures and ocular immune responses, functioning not only as a prevalent clinical condition but also as a sensitive indicator of environmental health. By integrating epidemiological and mechanistic evidence, this review highlights the relevance of environmental drivers in shaping ocular allergic disease and underscores the need for coordinated clinical and public health strategies to mitigate environmentally driven risk.

## Figures and Tables

**Table 1:** Characteristics of the included studies.

Author (Year)	Country	Design	Sample/Data Source	Main Exposure(s)	Outcome(s)	Key Finding(s)/Effect	MINORS
Akcay Usta & Icoz (2024) [25]	Turkey	Prospective observational	30 hazelnut harvesters	Occupational pollen exposure	Ocular surface disease	Hazelnut pollen exposure associated with ocular allergy and systemic inflammation	11
Anderson et al. (1997) [26]	UK	Clinical, biopsy	20 SAC patients + 16 controls	Grass pollen season	SAC histology	Mast cell–driven inflammation in SAC	18
Bhujel et al. (2024) [27]	South Korea	Experimental (animal)	OVA-sensitized mice ± PM	PM + OVA	Allergic eye disease	PM worsened OVA-induced AED via NF-κB and barrier disruption	22
Calderón (2024) [28]	Peru	Cross-sectional	80 patients	*Tipuana tipu* pollen	Rhinitis/conjunctivitis	New allergenic pollen identified in urban Lima	10
Chen et al. (2021) [29]	China	Time-series	99,276 visits	PM_2.5_, PM_10_, NO_2_, SO_2_	Conjunctivitis visits	Acute effects; NO_2_ strongest; sex-specific vulnerability	16
Chico-Fernández & Ayuga-Téllez (2024) [30]	Spain	Ecological time-series	Primary care records	Pollens + O_3_, PM, NO_2_, CO	AC & rhinitis	Synergistic pollen–pollutant effects	17
Chico-Fernández & Ayuga-Téllez (2025) [31]	Spain	Ecological time-series	Asthma care episodes	Pollens + pollutants	Asthma (related allergy)	O_3_ and pollen synergy	17
Eperon et al. (2004) [32]	Switzerland	Clinical observational	11 SAC patients + controls	Grass pollen season	Tear biomarkers	Eotaxin-1 as SAC activity marker	18
Ezinne et al. (2025) [33]	Trinidad & Tobago	Cross-sectional survey	591 adults	Pollen, mites, smoke, dust	OA prevalence	OA prevalence > 40%; Saharan dust influence	11
Gui et al. (2023) [34]	China	Time-series (DLNM)	59,731 visits	PM, NO_2_, O_3_, CO	Conjunctivitis visits	Stronger effects in infants & elderly; warm season	13
Gupta et al. (2025) [35]	India	Cross-sectional + cohort	8231 children	Sunlight, dust, smoke	VKC prevalence	VKC > 1%; strong geographic variability	23
Hong et al. (2016) [6]	China	Registry time-series	3.2 million visits	NO_2_, O_3_, PM, climate	AC visits	Lagged effects; NO_2_ & O_3_ predictors	13
Huang et al. (2024) [36]	China	Cross-sectional	131,304 children	PM_2.5_-bound CPs	AC symptoms	Higher risk in overweight children	18
Leonardi et al. (2015) [37]	Italy	Nationwide survey	3545 patients	Environmental triggers	OA diagnosis	Underuse of allergy testing	12
Levanon et al. (2023) [38]	Israel	Case-crossover	6024 VKC cases	Pollutants + climate	VKC flares	Higher risk in children and females	13
Liu et al. (2024) [39]	China	Time-series	3325 visits	PM, NO_2_, O_3_	AC visits	Lagged pollutant effects in arid climate	12
Lu et al. (2019) [40]	China	Multi-city case-crossover	81,351 visits	PM, NO_2_, O_3_	Conjunctivitis visits	Consistent pollution effects	13
MacLeod et al. (1997) [41]	UK	Clinical, biopsy	25 subjects	Grass pollen	SAC pathology	Mast cells as cytokine reservoirs	9
Mimura et al. (2014) [42]	Japan	Time-series	3002 visits	PM_2.5_	AC visits	Stronger effects outside pollen season	14
Mimura et al. (2024) [43]	Japan	Time-series	30,749 visits	SPM, NOx, Ox	AC diagnosis	SPM major determinant	15
Miyazaki et al. (2019) [44]	Japan	Nationwide survey	3004 respondents	NO_2_, PM_10_, Ox	SAC, PAC, VKC	Strongest ORs for VKC (NO_2_)	7
Nivenius et al. (2012) [45]	Sweden	Clinical CPT	21 subjects	Birch/grass pollen	AKC response	Early and late IgE-mediated responses	13
Phiri et al. (2025) [46]	Taiwan	Cross-sectional	136 children	Indoor + PM_10_	AC diagnosis	Effects stronger in boys	10
Singh et al. (2010) [47]	USA	Cross-sectional (NHANES)	20,010 adults	Weed pollens	OA prevalence	First national OA estimate	10
Tang et al. (2019) [48]	Taiwan	Experimental (animal)	ICR mice	PM_2.5_ eye drops	AC phenotype	PM_2.5_ directly induces AC	14
Qin et al. (2025) [49]	China	Experimental (animal + in vitro)	BALB/c mice, BMDMs	PM_2.5_	AC inflammation	TSLP & macrophage polarization	14
Qiu et al. (2024) [50]	China	Multi-city case-crossover	130,093 visits	O_3_	AC visits	~8% increase per IQR O_3_	14
Yanagisawa et al. (1999) [51]	Japan	Field occupational	22 farmers	Pear pollen	SAC symptoms	Occupational pollen induces SAC	13
Zhang et al. (2025) [52]	China	Experimental (RNA-seq)	BALB/c mice	Ragweed pollen	AC transcriptomics	lncRNAs implicated in AC	9

AC: allergic conjunctivitis; AED: allergic eye disease; AKC: atopic keratoconjunctivitis; BALB/c: BALB/c inbred mouse strain; BMDMs: bone marrow–derived macrophages; CPT: conjunctival provocation test; CPs: chlorinated paraffins; CO: carbon monoxide; DLNM: distributed lag non-linear model; IgE: immunoglobulin E; IQR: interquartile range; lncRNAs: long non-coding RNAs; MINORS: Methodological Index for Non-Randomized Studies; NHANES: National Health and Nutrition Examination Survey; NO_2_: nitrogen dioxide; NOx: nitrogen oxides; O_3_: ozone; OA: ocular allergy; OR: odds ratio; OVA: ovalbumin; Ox: photochemical oxidants; PAC: perennial allergic conjunctivitis; PM: particulate matter; PM_2.5_: particulate matter with aerodynamic diameter ≤ 2.5 µm; PM_10_: particulate matter with aerodynamic diameter ≤ 10 µm; RNA-seq: RNA sequencing; SAC: seasonal allergic conjunctivitis; SO_2_: sulfur dioxide; SPM: suspended particulate matter; TSLP: thymic stromal lymphopoietin; UK: United Kingdom; USA: United States of America; VKC: vernal keratoconjunctivitis.

## Data Availability

The original contributions presented in this study are included in the article/Supplementary Materials. Further inquiries can be directed to the corresponding author.

## Supplementary Materials

The following supporting information can be downloaded at: https://www.mdpi.com/article/10.3390/life16020271/s1, Supplementary File S1: PRISMA 2020 checklist; Supplementary File S2: Full electronic database search strategies (PubMed, Web of Science, Scopus); Supplementary File S3: Assessment of the quality of studies through Methodological Index for Non-Randomized Studies (MINORS); Supplementary File S4: Baseline characteristics of the 29 included studies.
