# Supplementary material for: Air Pollution, Pollen, and Indoor Exposures in Allergic Conjunctivitis: A Systematic Review
Source: Life (Basel). 2026 Feb 4;16(2):271. doi: 10.3390/life16020271 (PMC12941877; doi:10.3390/life16020271)
Supplement: Supplementary file 1 [file life-16-00271-s001.zip › Supplementary file S2.pdf]

Supplemental File S2. Full electronic search strategies.

Pubmed

Search: **(("Allergic conjunctivitis" OR "Ocular allergy" OR "Seasonal allergic conjunctivitis" OR "Perennial allergic conjunctivitis" OR "Vernal keratoconjunctivitis" OR "Atopic keratoconjunctivitis") AND ("Climate change" OR "Global warming" OR "Air pollution" OR "Atmospheric pollution" OR "Particulate matter" OR PM2.5 OR PM10 OR "Pollen" OR "Aeroallergens" OR "Ultraviolet radiation" OR "Environmental exposure"))** Sort by: **Most Recent**

("Allergic conjunctivitis"[All Fields] OR "Ocular allergy"[All Fields] OR "Seasonal allergic conjunctivitis"[All Fields] OR "Perennial allergic conjunctivitis"[All Fields] OR "Vernal keratoconjunctivitis"[All Fields] OR "Atopic keratoconjunctivitis"[All Fields]) AND ("Climate change"[All Fields] OR "Global warming"[All Fields] OR "Air pollution"[All Fields] OR "Atmospheric pollution"[All Fields] OR "Particulate matter"[All Fields] OR "PM2.5"[All Fields] OR "PM10"[All Fields] OR "Pollen"[All Fields] OR "Aeroallergens"[All Fields] OR "Ultraviolet radiation"[All Fields] OR "Environmental exposure"[All Fields])

Web of Science

TS=("Allergic conjunctivitis" OR "Ocular allergy" OR "Seasonal allergic conjunctivitis" OR "Perennial allergic conjunctivitis" OR "Vernal keratoconjunctivitis" OR "Atopic keratoconjunctivitis")

AND

TS=("Climate change" OR "Global warming" OR "Air pollution" OR "Atmospheric pollution" OR "Particulate matter" OR "PM2.5" OR "PM10" OR "Pollen" OR "Aeroallergens" OR "Ultraviolet radiation" OR "Environmental exposure")

Scopus

TITLE-ABS-KEY("Allergic conjunctivitis" OR "Ocular allergy" OR "Seasonal allergic conjunctivitis" OR "Perennial allergic conjunctivitis" OR "Vernal keratoconjunctivitis" OR "Atopic keratoconjunctivitis")

AND

TITLE-ABS-KEY("Climate change" OR "Global warming" OR "Air pollution" OR "Atmospheric pollution" OR "Particulate matter" OR "PM2.5" OR "PM10" OR "Pollen" OR "Aeroallergens" OR "Ultraviolet radiation" OR "Environmental exposure")
