# Supplementary material for: Air Pollution, Pollen, and Indoor Exposures in Allergic Conjunctivitis: A Systematic Review
Source: Life (Basel). 2026 Feb 4;16(2):271. doi: 10.3390/life16020271 (PMC12941877; doi:10.3390/life16020271)
Supplement: Supplementary file 1 [file life-16-00271-s001.zip › Supplementary file S3 .pdf]

Supplementary file S3 : Assessment of the quality of studies through Methodological Index for Non-Randomized Studies (MINORS).

Study	Clearly stated aim	Consecutive patients	Prospective collection data	Endpoints	Assessment endpoint	Follow-up period	Loss less than 5%	Study size	Adequate control group	Contemporary group	Baseline control	Statistical analyses	MINORS
Akcay Usta and Icoz (2024) [25]	2	1	1	2	2	1	0	2	-	-	-	-	11
Anderson et al. (1997) [26]	2	1	1	2	2	0	2	1	2	2	1	2	18
Bhujel et al. (2024) [27]	2	2	2	2	1	2	2	1	2	2	2	2	22
Calderón (2024) [28]	2	1	1	2	1	0	2	1	-	-	-	-	10
Chen et al. (2020) [29]	2	2	1	2	1	2	2	2	0	0	0	2	16
Chico-Fernández and Ayuga-Téllez (2024) [30]	2	2	1	2	1	2	2	2	0	1	0	2	17
Chico-Fernández and Ayuga-Téllez (2025) [31]	2	2	1	2	1	2	2	2	0	1	0	2	17
Eperon et al. (2004) [32]	2	1	1	2	2	0	2	1	2	2	1	2	18
Ezinne et al. (2025) [33]	2	1	1	2	1	0	2	2	-	-	-	-	11
Gui et al. (2023) [34]	2	2	0	2	1	2	2	2	-	-	-	-	13
Gupta et al. (2025) [35]	2	2	2	2	2	2	1	2	2	2	2	2	23
Hong et al. (2016) [6]	2	2	0	2	1	2	2	2	-	-	-	-	13
Huang et al. (2024) [36]	2	2	1	1	1	0	2	2	2	2	1	2	18
Leonardi et al. (2015) [37]	2	2	1	2	1	0	2	2	-	-	-	-	12
Levanon et al. (2023) [38]	2	2	0	2	1	2	2	2	-	-	-	-	13
Liu et al. (2024) [39]	2	2	0	1	1	2	2	2	-	-	-	-	12
Lu et al. (2019) [40]	2	2	0	2	1	2	2	2	-	-	-	-	13
Macleod et al. (1997) [41]	2	0	1	2	1	0	2	1	-	-	-	-	9
Mimura et al. (2014) [42]	2	2	1	2	1	2	2	2	-	-	-	-	14
Mimura et al. (2024) [43]	2	2	2	2	1	2	2	2	-	-	-	-	15
Miyazaki et al. (2019) [44]	2	0	0	2	0	0	1	2	-	-	-	-	7

Nivenius et al. (2012) [45]	2	1	2	2	1	2	2	1	-	-	-	-	13
Phiri et al. (2025) [46]	2	1	1	2	1	0	2	1	-	-	-	-	10
Singh et al. (2010) [47]	2	2	0	2	1	0	1	2	-	-	-	-	10
Tang et al. (2019) [48]	2	2	2	2	1	2	2	1	-	-	-	-	14
Qin et al. (2025) [49]	2	2	2	2	1	2	2	1	-	-	-	-	14
Qiu et al. (2024) [50]	2	2	2	2	1	2	2	1	-	-	-	-	14
Yanagisawa et al. (1999) [51]	2	2	0	2	1	2	2	2	-	-	-	-	13
Zhang et al. (2025) [52]	2	1	0	2	1	0	2	1	-	-	-	-	9
