# Supplementary material for: Air Pollution, Pollen, and Indoor Exposures in Allergic Conjunctivitis: A Systematic Review
Source: Life (Basel). 2026 Feb 4;16(2):271. doi: 10.3390/life16020271 (PMC12941877; doi:10.3390/life16020271)
Supplement: Supplementary file 1 [file life-16-00271-s001.zip › Supplementary file S4.pdf]

Supplementary file S4. Baseline characteristics of the 29 included studies.

Author (Year)	Study Type	Exposure	Population / Model	Notes
Akcay Usta and Icoz (2024) [25]	Prospective observational	Hazelnut harvesting season (pollen, occupational exposure)	30 hazelnut harvesters with moderate ocular surface disease	Suggests hazelnut pollen exposure induces ocular allergy with systemic inflammatory involvement; potential role of blood biomarkers as indicators of ocular surface disease severity.
Anderson et al. (1997) [26]	Clinical, conjunctival biopsy with immunohistochemistry	Seasonal allergic conjunctivitis (grass pollen season vs out of season)	20 SAC patients (8 in-season, 12 out-of-season) and 16 controls, UK	First direct histological demonstration that SAC is mast cell–driven without major granulocyte infiltration, distinguishing it from VKC/AKC.
Bhujel et al. (2024) [27]	Experimental (animal, AED mouse model)	Particulate matter (PM, SRM 2786, <4 µm) co-exposure with ovalbumin (OVA)	C57BL/6 mice, OVA-sensitized, challenged ± PM exposure	Demonstrates adjuvant role of PM in worsening allergic eye disease (AED). Highlights NF-κB activation, disruption of ocular surface integrity, systemic allergic response.
Calderón (2024) [28]	Cross-sectional / Aerobiological	Pollen (*Tipuana tipu*)	80 patients with rhinitis/conjunctivitis in Lima (18–50 years)	New environmental allergen described; aerobiology with Burkard trap (2020–2021)
Chen et al. (2020) [29]	Time-series, hospital outpatient data (2015–2019)	Air pollutants: PM2.5, PM10, SO_2, NO_2	99,276 conjunctivitis visits, stratified by age, sex, occupation	Effects were acute (same day to 2 days). NO_2 strongest risk. Gender differences: males more sensitive to NO_2, females to SO_2. Recommends protecting vulnerable populations during high pollution.
Chico-Fernández and Ayuga-Téllez (2024) [30]	Ecological / time-series analysis (CAM, Spain)	Six air pollutants (O_3, PM10, PM2.5, NO_2, CO, SO_2) + six tree pollens (Cupressaceae, Olea, Platanus, Pinus, Ulmus, Populus)	Health records of primary care (rhinitis & allergic conjunctivitis) linked to Palinocam pollen data and Air Quality Networks (2014–2017)	Urban ecological study; emphasizes synergistic effects of pollen + pollutants. Suggests planning urban green spaces to avoid highly allergenic species.
Chico-Fernández and Ayuga-Téllez (2025) [31]	Ecological / Time-series (CAM, Spain)	Six atmospheric pollutants (O_3, PM10, PM2.5, NO_2, CO, SO_2) + six tree pollens (Cupressaceae, Olea, Platanus, Pinus, Ulmus, Populus)	Daily asthma care episodes (primary care electronic records, ICPC-2 code R96) linked to Palinocam pollen data and CAM Air Quality Network (2014–2017)	Shows synergistic effect of pollen + pollutants on asthma. Suggests urban green planning with allergenicity in mind; highlights role of O_3.
Eperon et al. (2004) [32]	Clinical, observational (tears + ELISA)	Seasonal allergic conjunctivitis during grass pollen season (vs out of season, vs controls)	11 SAC patients, 6 controls; 5 patients sampled in and out of season	Confirms role of eotaxin-1 as biomarker of SAC activity; supports link with eosinophil recruitment via CCR3.
Ezinne et al. (2025) [33]	Cross-sectional, web-based survey	Environmental exposures: pollen, mites, cigarettes, air conditioning; systemic comorbidities: asthma, allergic rhinitis, eczema	591 adults (≥18 years), 74% female, mean age 18–24 yrs (50%)	First OA prevalence study in Caribbean adults. Warm climate + Sahara dust may increase burden. Awareness low; recommends screening OA in patients with asthma/rhinitis.
Gui et al. (2023) [34]	Time-series, quasi-Poisson + DLNM, 2013–2020	PM2.5, PM10, SO_2, NO_2, CO, O_3	59,731 outpatient conjunctivitis visits (largest ophthalmology clinic in Xinjiang)	Strongest associations: NO_2 in newborns (0–1 y) and elderly (≥65 y), PM10 in 6–64 y/o, CO in females and 6–64 y/o. Warm season showed

				stronger associations for most pollutants. SO_2 reductions in Urumqi linked to ↓ risk, showing effectiveness of control policies. Sub-analyses by conjunctivitis subtype: PM and NO_2 → acute/chronic; CO → allergic/non-specific.
Gupta et al. (2025) [35]	Cross-sectional + cohort (multicentric, India)	Environmental factors: bright sunlight, dust/pollution, smoke from incense/mosquito coils, windy weather, seasonal influences (spring, winter). Physical environmental parameters (UVA flux).	8,231 children/adolescents (5–15 years) from rural and urban sites across northern plains, hilly NE, high-altitude, and coastal India	Large multicentric community-based study; included nested cohort follow-up (10 years). Rural prevalence of VKC > urban. Strong geographic variation: highest in northern plains, lowest in southern coastal.
Hong et al. (2016) [6]	Retrospective registry, time-series (2008–2012)	Ambient air pollutants (NO_2, O_3, PM10, PM2.5, SO_2), temperature, humidity, wind velocity	3,211,820 outpatient visits for allergic conjunctivitis (Shanghai Health Insurance System)	Large-scale dataset, time-lag effect (~3 weeks) observed. Limitations: no pollen data, potential selection bias, registry-based diagnosis.
Huang et al. (2024) [36]	Cross-sectional epidemiological study	PM2.5-bound chlorinated paraffins (SCCPs, MCCPs, LCCPs)	131,304 children and adolescents (Pearl River Delta, China)	Stronger associations in overweight/obese children; SCCPs showed highest risk; evidence of immunotoxicity of CPs
Leonardi et al. (2015) [37]	Cross-sectional, nationwide survey (Italy)	Clinical ocular allergy (questionnaire-based, triggers and treatments)	3,545 patients (mean age 38 yrs) recruited by 304 ophthalmologists across Italy	One of the largest national surveys of ocular allergy; highlights underuse of allergy testing (35%) and mismatch between treatment practices and guidelines. Provides demographic, clinical, and pharmacological landscape of ocular allergy in Europe.
Levanon et al. (2023) [38]	Retrospective, population-based, case-crossover	Air pollutants (NO_2, O_3, PM10, PM2.5, SO_2), meteorological (temperature, solar radiation, RH)	6,024 VKC patients, Southern Israel (2000–2021)	First large-scale epidemiological study linking VKC flares to multiple environmental factors. Stratified results show higher risk in children, females, Jews for O_3, Bedouins for PM10. Suggests prevention strategies (behavioral adaptation, protective eyewear).
Liu et al. (2024) [39]	Time-series (quasi-Poisson + DLNM, 2013–2020)	Six major pollutants (PM2.5, PM10, NO_2, SO_2, CO, O_3)	3,325 allergic conjunctivitis outpatient visits, Urumqi (NW China)	First long-term study in western China; highlights lagged effects of pollutants; unique setting (arid climate, high PM10 from coal, sandstorms).
Lu et al. (2019) [40]	Multi-city epidemiological study (time-stratified case-crossover)	Short-term air pollution: PM2.5, PM10, NO_2, SO_2, O_3 (per 10 μg/m³ increase)	81,351 conjunctivitis outpatients (2013–2014) in 4 Chinese cities (Qingdao, Zhengzhou, Chongqing, Guangzhou)	First multi-city Chinese study; confirms role of air pollution in ocular surface disease; possible harvesting effect; limitations: hospital data only, fixed monitoring exposure, no subtype classification of conjunctivitis.

Macleod et al. (1997) [41]	Clinical, conjunctival biopsy + immunohistochemistry	Seasonal allergic conjunctivitis (grass pollen season vs. out of season)	8 SAC patients in-season, 8 out-of-season, 9 non-allergic controls (UK)	Shows mast cells as key regulators of ocular allergy, not only effector cells but also cytokine reservoirs; IL-4 release drives upregulation of allergic inflammation.
(Mimura et al. (2014) [42]	Time-series analysis (non-pollen & pollen seasons)	PM2.5 (fine particulate matter)	3,002 allergic conjunctivitis outpatients, Tokyo (May–Oct 2012)	First Japanese hospital-based study linking PM2.5 to AC; suggests stronger effect outside pollen season
Mimura et al. (2024) [43]	Observational (time-series, prospective clinic-based)	Suspended Particulate Matter (SPM), Ox, NOx, CO, weather factors	30,749 outpatient visits in Tokyo (6,145 diagnosed with allergic conjunctivitis, 2012)	Suggests SPM is a major determinant of allergic conjunctivitis exacerbations; stronger impact in winter/spring; possible role as adjuvant/allergen carrier.
Miyazaki et al. (2019) [44]	Nationwide cross-sectional, web-based survey (2017)	Ambient air pollution: NO, NO_2, NOx, oxidants, SO_2, $PM_{2}.5$, PM_{10} (2012–2016 data)	3,004 respondents (ophthalmologists & family members, nationwide Japan)	Prevalence: SAC 37.4%, PAC 14.0%, AKC 5.3%, VKC 1.2%. VKC association strongest (NO_2 OR=1.88, PM_{10} OR=1.54). SO_2 mostly null. $PM_{2}.5$ inversely related to AKC (OR=0.81). Highlights TRAPs (traffic-related pollutants) as risk factors for severe ocular allergy. Funding: Santen Pharmaceutical (no role in design/analysis).
Nivenius et al. (2012) [45]	Clinical study, conjunctival provocation test (CPT)	Airborne allergens (birch or grass pollen, single-dose instillation)	11 AKC patients, 5 SAC patients, 5 healthy controls (Sweden)	First CPT in AKC; demonstrates both early-phase and late cytokine responses. Confirms AKC as chronic IgE-driven but with T-cell/cytokine involvement.
Phiri et al. (2025) [46]	Cross-sectional (LEAPP-HIT birth cohort, Taiwan)	Indoor exposures (new furniture, carpets, moldy smells, anti-bug products, endotoxins, Der f 1); Outdoor exposures (PM10, POI: fast-food restaurants, night markets, waste recycling)	136 children <5 years, Greater Taipei Area	Stronger associations in boys (allergic conjunctivitis reported only in males); h
Singh et al. (2010) [47]	Cross-sectional (NHANES III, 1988–1994)	Self-reported ocular/nasal allergy symptoms + skin prick testing	20,010 US adults	First national epidemiological estimate of ocular allergy in US. Seasonal peaks: ocular (June–July), nasal (spring/fall). Skin testing showed strongest associations with weed pollens. EPA STAR grant link to climate change research.
Tang et al. (2019) [48]	Experimental (animal model, ICR mice, 19 days)	PM2.5 (3.2, 6.4, 12.8 mg/mL) eye drops	50 female ICR mice, direct ocular exposure	First murine AC model directly induced by PM2.5 exposure. Distinguishes AC from dry eye. Artificial tears did not alleviate symptoms. Highlights mechanistic role of PM2.5 in ocular allergy.

Qin et al. (2025) [49]	Experimental, murine model + in vitro (BMDMs)	PM2.5 (sensibilización intraplantar + desafío tópico ocular; 25 µg/mL en BMDMs)	BALB/c mice (PM2.5-induced AC) + bone marrow–derived macrophages	First reproducible PM2.5-induced AC model. Shows macrophage polarization to M1 and identifies TSLP as central mediator linking PM2.5 exposure to ocular allergy. Supported by NSFC & Shaanxi Key R&D grants.
Qiu et al. (2024) [50]	Multi-city, time-stratified case-crossover	Ozone (O_3)	130,093 outpatient visits for allergic conjunctivitis across 5 Chinese cities (2014–2022)	Adjusted for PM2.5, CO, SO_2, NO_2; consistent across sensitivity analyses
Yanagisawa et al. (1999) [51]	Observational, field study (orchard workers)	Japanese pear (Pyrus pyrifolia) pollen	22 farmers performing artificial pollination in pear orchards	Symptoms resolved with topical cromoglycate. Demonstrated that *entomophilous* flower pollen (pear) can induce SAC. Small sample, occupational exposure.
(Zhang et al. (2025) [52]	Experimental, transcriptomic (RNA-seq)	Ragweed pollen-induced allergic conjunctivitis	BALB/c mice (AC model vs controls)	First study profiling immune-related lncRNAs in ocular allergy. Suggests mucosal immunity molecules may act as protective/ modulatory factors in AC. Provides potential new therapeutic targets.

AED: Allergic Eye Disease; AKC: Atopic Keratoconjunctivitis; CCR3: CC Chemokine Receptor 3; CO: Carbon Monoxide; CPT: Conjunctival Provocation Test; Der f 1: Dermatophagoides farinae 1 (dust mite allergen); DLNM: Distributed Lag Non-linear Model; ELISA: Enzyme-Linked Immunosorbent Assay; lncRNA: Long Non-coding RNA; NF-κB: Nuclear Factor Kappa-light-chain-enhancer of Activated B Cells; NHANES: National Health and Nutrition Examination Survey; NO_2: Nitrogen Dioxide; NOx: Nitrogen Oxides; OA: Ocular Allergy; O_3: Ozone; OVA: Ovalbumin; Ox: Oxidants; PAC: Perennial Allergic Conjunctivitis; PM: Particulate Matter; $PM_{2.5}$ / PM_{10}: Particulate Matter <2.5 µm / <10 µm; RH: Relative Humidity; SAC: Seasonal Allergic Conjunctivitis; SCCPs / MCCPs / LCCPs: Short-, Medium-, and Long-chain Chlorinated Paraffins; SO_2: Sulfur Dioxide; SPM: Suspended Particulate Matter; TSLP: Thymic Stromal Lymphopoietin; UVA: Ultraviolet A; VKC: Vernal Keratoconjunctivitis.
